# Functional Redundancy and Complementarities of Seed Dispersal by the Last Neotropical Megafrugivores

**DOI:** 10.1371/journal.pone.0056252

**Published:** 2013-02-07

**Authors:** Rafael S. Bueno, Roger Guevara, Milton C. Ribeiro, Laurence Culot, Felipe S. Bufalo, Mauro Galetti

**Affiliations:** 1 Laboratório de Biologia da Conservação, Departamento de Ecologia, Universidade Estadual Paulista, Rio Claro, Sao Paulo, Brazil; 2 Instituto de Ecología, A.C. Departamento de Biología Evolutiva, Xalapa, Veracruz, Mexico; 3 Laboratório de Ecologia Espacial e Conservação, Departamento de Ecologia, Universidade Estadual Paulista, Rio Claro, Sao Paulo, Brazil; Institut Mediterrani d´Estudis Avançats (CSIC/UIB), Spain

## Abstract

**Background:**

Functional redundancy has been debated largely in ecology and conservation, yet we lack detailed empirical studies on the roles of functionally similar species in ecosystem function. Large bodied frugivores may disperse similar plant species and have strong impact on plant recruitment in tropical forests. The two largest frugivores in the neotropics, tapirs (*Tapirus terrestris*) and muriquis (*Brachyteles arachnoides*) are potential candidates for functional redundancy on seed dispersal effectiveness. Here we provide a comparison of the quantitative, qualitative and spatial effects on seed dispersal by these megafrugivores in a continuous Brazilian Atlantic forest.

**Methodology/Principal Findings:**

We found a low overlap of plant species dispersed by both muriquis and tapirs. A group of 35 muriquis occupied an area of 850 ha and dispersed 5 times more plant species, and 13 times more seeds than 22 tapirs living in the same area. Muriquis dispersed 2.4 times more seeds in any random position than tapirs. This can be explained mainly because seed deposition by muriquis leaves less empty space than tapirs. However, tapirs are able to disperse larger seeds than muriquis and move them into sites not reached by primates, such as large forest gaps, open areas and fragments nearby. Based on published information we found 302 plant species that are dispersed by at least one of these megafrugivores in the Brazilian Atlantic forest.

**Conclusions/Significance:**

Our study showed that both megafrugivores play complementary rather than redundant roles as seed dispersers. Although tapirs disperse fewer seeds and species than muriquis, they disperse larger-seeded species and in places not used by primates. The selective extinction of these megafrugivores will change the spatial seed rain they generate and may have negative effects on the recruitment of several plant species, particularly those with large seeds that have muriquis and tapirs as the last living seed dispersers.

## Introduction

It has been hypothesized that in biodiversity-rich ecosystems, the extinction of some species will not cause a substantial loss in ecosystem function if remnant species play equivalent roles and are capable of taking over the functions played by extinct species (i.e. functional redundancy) [1]. Analysis of the seed dispersal network in tropical forests shows high connectedness and diet overlap among several species and groups of vertebrates [2], suggesting high redundancy in the system. The selective and relatively fast removal in the diversity and biomass of large-bodied vertebrates, a phenomenon named “defaunation” [3], is creating shifts in vertebrate communities often dominated now by few small-bodied species [4], [5], with potential changes in their functional diversity in the vertebrate communities. Erosion of functional diversity via defaunation may ultimately affect differently the recruitment of plant species, depending on functional traits such as fruit and seed sizes [6], [7], [8], [9].

Large vertebrates are particularly important, because they remove a larger amount of seeds, disperse them for longer distances, and are able to disperse larger seeds than smaller frugivores [10], [11], [12], [13]. Primates play an important role in forest dynamics, as they are the largest arboreal forest frugivores and constitute 25%–40% of the frugivore biomass in most tropical forests [14]. Ateline primates (e.g. *Ateles, Brachyteles, Logothrix*), for instance, can disperse millions of seeds per year [15], and thanks to their wide variety of feeding behaviors, they create different seed shadows and dispersal kernels [11], [16]. Another important fruit-eating group in the neotropics is large ungulates, such as peccaries, deer and tapirs, which comprises the largest extant frugivores in tropical forests. While peccaries and deer are most seed predators, tapirs (*Tapirus* spp.) eat large amounts of fruits, disperse large quantities of seeds, often at long distances, and are able to disperse very large seeds even across heavily disturbed areas [17], [18], [19]. These megafrugivores are amongst the most hunted animals in the neotropics. For instance every year about 47,000 tapirs and 700,000 ateline monkeys are killed in the Amazon forest for subsistence [20]. The situation is no better in the Atlantic forest, where only 12% of the original forest is left [21] and hunting is still widespread [22].

**Figure 1:**
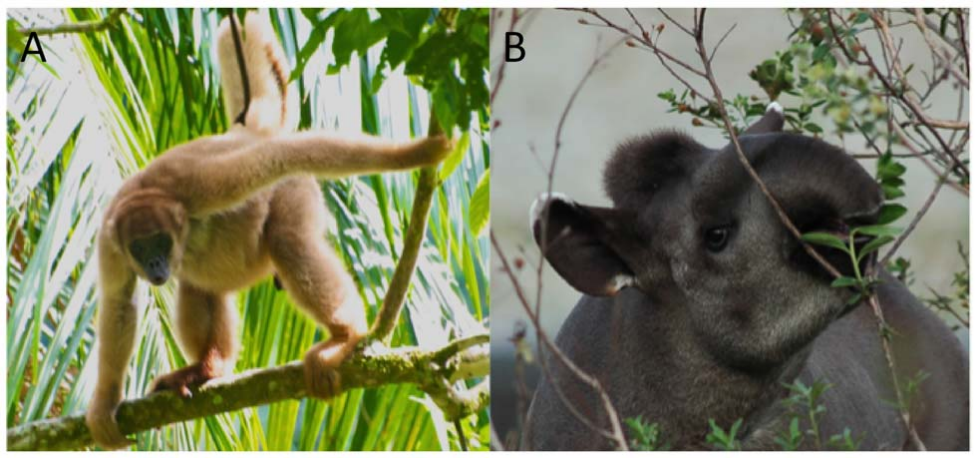
The largest arboreal and terrestrial frugivore in the Neotropics: the muriqui (*Brachyteles arachnoides*) (A) and the tapir (*Tapirus terrestris*) (B). Photos by Pedro Jordano and Mauro Galetti, respectively.

Neotropical rainforests may be particularly sensitive to the removal of frugivores because between 40% and 90% of woody species bear fleshy fruits dispersed by vertebrates [23]. Although seed dispersal by primates and ungulates has been widely studied in terms of seed dispersal [24], [25], [26], we lack comparative studies on seed dispersal effectiveness between co-occurring large-bodied species. Are the largest megafrugivores, tapirs and ateline primates, redundant or complementary seed dispersers? This question is particularly important if we want to predict the impact of defaunation on plant recruitment [27] and ecosystem function. Here we compare the seed dispersal effectiveness of the largest remaining arboreal and terrestrial frugivores in the Neotropical forests, the muriqui (*Brachyteles arachnoides*) and the tapir (*Tapirus terrestris*) (Figure 1).

## Methods

### Study Site

**Figure 2:**
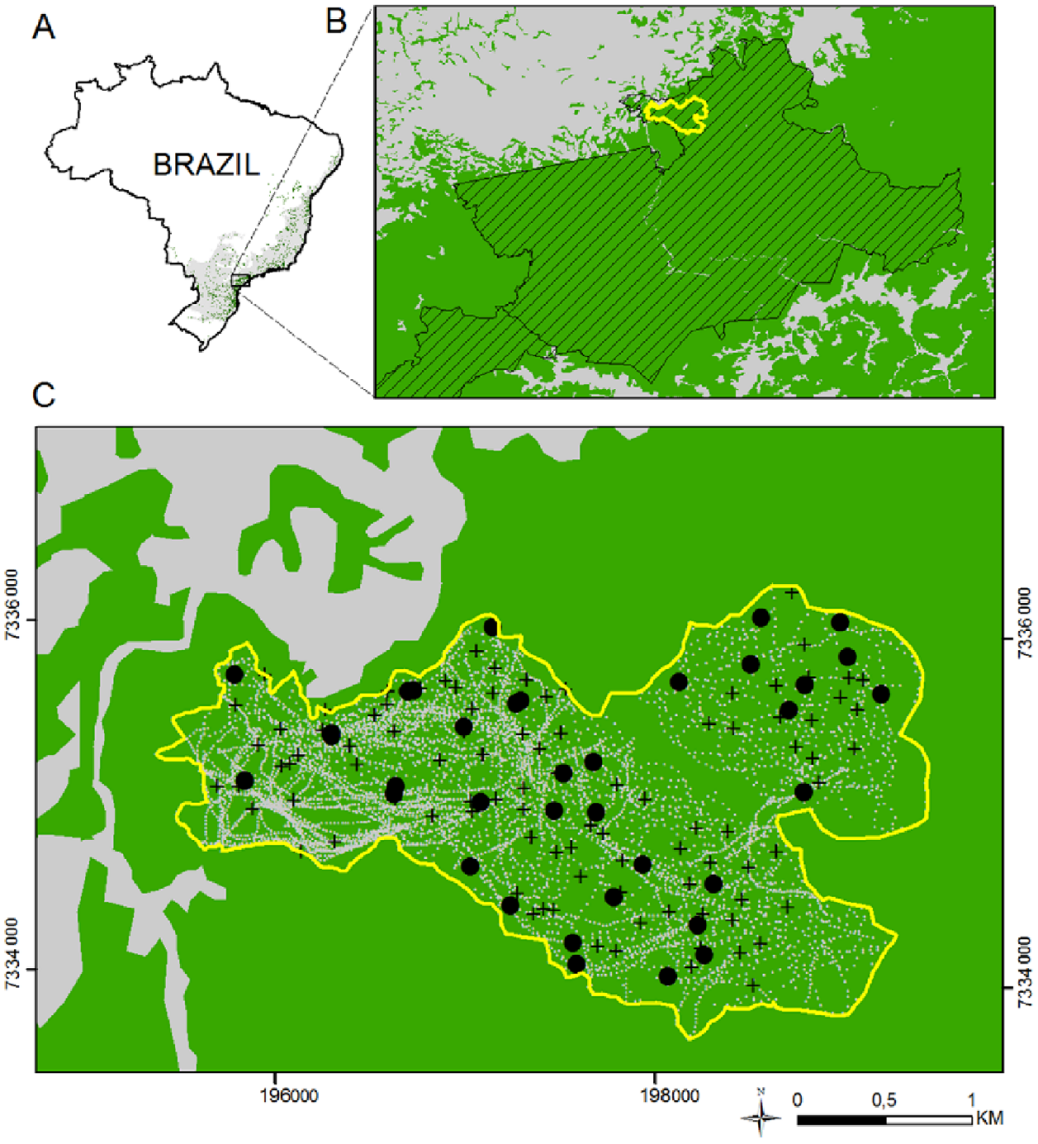
Location of the study area in the Atlantic forest, Brazil. (A) Historic distribution (in gray) and actual remnants (in green) of the Atlantic forest; (B) Location of the studied area (in yellow) for group of muriquis (*Brachyteles arachnoides*) in Carlos Botelho State Park (hatched area); (C) routes of the muriquis (gray dotted lines) and defecations of muriquis (+) and tapirs (*Tapirus terrestris*)(black dots). Green is forest remnants and light gray is open matrix (pastures) in B and C.

This study was carried out at Carlos Botelho State Park (hereafter CBSP), a 37,644 ha Protected Area in a continuous massif named “Serra de Paranapiacaba” in São Paulo state, southeastern Brazil (24°44′ S, 47°44′ W; Figure 2). The topography is hilly, with slopes ranging from 10° up to 50° degrees, and very few flat tracts, usually along larger rivers. There is a high altitudinal variation inside CBSP, ranging from 30 m to 1100 m a.s.l. Average rainfall is about 2,000 mm/year, with no clear dry season and mean temperature is 20°C. The vegetation is Ombrophilous Dense Atlantic Forest, ranging from lowland to montane physiognomy [28]. More than 1151 species of woody plants have been recorded, with the most abundant families represented by Myrtaceae, Arecaceae, Lauraceae, Rubiaceae, Sapotaceae and Moraceae [29]. The landscape around the park is dominated by monocultures of *Pinus* and *Eucalyptus*, pastures, and other small-scale agricultural crops. The Paranapiacaba massif comprises more than 120,000 ha of Atlantic Forest distributed through four protected parks and several private properties and holds an almost complete assemblage of threatened mammals, including jaguars (*Panthera onca*), bush dogs (*Speothos venaticus*), tapirs (*Tapirus terrestris*) and muriquis (*Brachyteles arachnoides*), although white-lipped peccaries (*Tayassu pecari*) are locally extinct [22], [30]. Specifically, our study was conducted in the home range of a group of 35 southern muriquis *Brachyteles arachnoides* (hereafter muriquis), corresponding to approximately 850 ha at about 750 m a.s.l, located at the extreme north of CBSP [31] (Figure 2).

### Feces Deposition Pattern and Seed Dispersal

From April 2008 to July 2009, we searched for tapir feces by walking along a grid of existing trails and also following tapirs tracks distributed throughout the study area, totaling 198 km walked, with an average of 14 km per month. The effort was distributed in such a way that all topographic features (small rivers, dry valleys, slope and top hill) were sampled in equal proportions. We defined as “latrine” a clump of tapir feces with different ages inside a 5-meter radius [25]. The latrines were visited monthly and we collected dispersed seeds in one third of the whole fecal material in order to not interfere in the use of the latrine, so to quantify the seed dispersal we multiplied the results of the analyzed samples by three.

During the same period, we followed individuals of the sub-groups of muriquis habituated to human presence, from sunrise (5∶15 h) to sunset (18∶30 h) totaling 246,7 km walked with an average of 17,5 km per month. Observations totaled 432 h of direct contact, of which 382 h (89%) were on adults. We totaled 31 complete days of observation following the same sub-group from sunrise (waking-up site) to sunset (sleeping site) and 20 incomplete days (i.e. observation interrupted for more than 1 hour). To verify the sub-group size we collected data on the number of muriquis surrounding the focal individual (about 15 m) three times a day (8∶00, 12∶00 and 16∶00). Points along daily routes were geopositioned every 15 minutes. To determine the feces deposition pattern, each observed defecation event, here defined as a defecation of a single individual at the same place within 1 minute, was also geopositioned. The deposition pattern was defined as “clumped” if the feces fell in less than 30 cm radius, or “scattered” if, while falling, the feces collided with the leaves and branches of the understory and reached the ground in more than 30 cm radius [26].

Muriqui and tapir feces were collected and analyzed in the laboratory. When tapir feces were located in water we only collected them if the boluses were whole (i.e. not broken or dissolved). For all samples, we recorded the topographical feature (water, dry valley, slope or hilltop), distance (m) from the nearest tree with DBH >40 cm and all possible parental fruiting trees in an area of 25 m radius. All feces were sieved through a mesh of 2×2 mm and all remaining seeds were identified with the aid of guides [32] and by comparing with a collection of voucher specimens. Seeds were also counted, measured with digital calipers with 0.01 mm precision and classified as “predated” if broken or chewed or “non predated” if no damage was observed [33]. The seeds ≤2 mm (e.g. *Coussapoa* sp.) were collected in a plate under the sieve and whenever possible were identified and the number of seeds estimated. We also collected fallen fruits under fruiting trees, removed the pulp and measured the seeds for comparison with dispersed seeds. To estimate the minimum seeds dispersed by month by muriquis we used the average of 30.6 seeds dispersed by day, obtained from five complete days where we were able to collect at least 4 defecation of the same individual (R. S. Bueno, unpublished data) and multiplied by 30 days. For tapirs, we used the average of 140 seeds per individual per month. This was based on the number of new seeds found per month in specific latrines that were monitored monthly.

### Germination Experiments

We conducted germination tests to determine the effect of the passage through tapir and muriqui guts on the germination success (germinated seeds/total seeds) and time to germination (average days for germination) of the seeds. Due to the difficulty in finding large enough quantities of seeds for the trials, we chose one small-seeded (*Hieronyma alchorneoides*, Phyllanthaceae) and one large-seeded species (*Cryptocarya mandioccana*, Lauraceae). The fruits collected on the ground or directly from trees were manually defleshed and seeds cleaned. For *C. mandioccana* we also used the whole fruits (i.e. with pulp). We just used seeds with no sign of insect damage or predation. The seeds of *H. alchorneoides* are dispersed by muriquis, tapirs, and small birds, while the seeds of *C. mandioccana* are dispersed by primates (muriquis and howlers monkeys), tapirs, and piping guans (*Aburria jacutinga*) [34], [35]. We sowed 100 seeds of *C. mandioccana*, 25 from tapir feces, 25 from muriqui feces, 25 seeds cleaned manually and 25 whole fruits (with pulp), and 75 of *H. alchorneoides* with the same methods as *C. mandioccana*, excepting whole fruits. The seeds were placed individually in plastic bags with sterilized soil and placed in a greenhouse located right at the border of the CBSP forest. The greenhouse is covered with a shadow mesh of 50% of sunlight retention and all seeds were watered and monitored daily.

### Statistical Analyses

To verify the redundancies and complementarities of the seed dispersal by tapirs and muriquis, here defined as the spatial (deposition pattern in space), quantitative (number of seeds dispersed) and qualitative (germination) overlap, we adopted the following criteria and indicators [36]: number of species dispersed, number of dispersed seeds per feces, size of dispersed seeds, pattern of feces deposition (clumped or scattered), rate and time for germination (measured in greenhouse), and spatial distribution of the feces. Since feces can be analyzed as a point-based pattern process, we used the empty space function to estimate the spacing between each event (feces) in order to test the individual and joint contribution of muriquis and tapirs on spatial seed dispersal. The lower the empty space is, the more widespread is the dispersal. The package *spatstat* available in R was used in this step [37]. We generated three empty space maps using *distmap* function for three situations: muriquis only; tapirs only; muriquis+tapirs. To understand the contribution of muriquis and tapirs to spatial seed dispersal, we distributed 1,000 random points on the area of interest using a Poisson-based distribution. For each random point, we estimated the empty space (i.e. distance to nearest feces) for the three situations mentioned above. T-tests were applied to test statistic significances between empty space estimates for paired seed disperser situations. Seed deposition quantity (i.e. the number of seeds within a determined radius per any random position) was estimated for muriquis, tapirs and muriquis+tapirs using interpolation methods available within *spatstat* R package. We estimated the number of seeds dispersed within a search radius of 250 m. This value was adopted because it is close to the average empty space accounted for feces deposition recorded for the species with the largest empty space (i.e. tapir). Student T-tests were applied to test statistic significances for seed deposition quantity for paired seed disperser situations.

To compare the intraspecific sizes of seeds dispersed by muriquis and tapirs, we used a nested ANOVA with disperser nested within the specific identity of the plant. The disperser factor had three levels, muriquis, tapirs (seeds collected from dung) and control, i.e., a random sample of seeds collected from trees and ground. We used post hoc t-test based on standard errors estimated from the linear predictor of the ANOVA model to uncover particular differences.

Germination velocity was analyzed through a Cox proportional hazards regression model [38] in which the change of stage was given by the germination of seeds. To deal with ties we used the Efron approximation, since it is accurate and computationally efficient.

To compare the overlap in number of species with different seed size we used a contingency table together with residual analysis and a randomization test to uncover particular significant differences across the table. Randomization was done within each seed size level.

## Results

### Frugivory and Seed Rain Generated by Muriquis and Tapirs

We analyzed 106 defecations of muriquis and 49 of tapirs. Muriquis dispersed 28 seed species, with an average of 23.3±17.5 (13–86) seeds larger than 2 mm per fecal sample, while tapirs dispersed six seed species, with an average of 72.5±48.8 (13–183) seeds larger than 2 mm per fecal sample. Muriquis dispersed 2.04±1.25 (mean ± SD) species with a maximum of five species in a single defecation, while tapirs dispersed 1.26±0.45 species with a maximum of 2 species in a single defecation. Several seed species smaller than 2 mm were deposited in a single defecation (e.g. *Coussapoa microcarpa,* Urticaceae with more than 80 seeds). Of all seeds found in tapir feces, just 12 seeds of *Cryptocarya mandioccana* (4.7%, n = 261) were mechanically predated. Two species were predated by muriquis (*Ocotea catharinensis,* Lauraceae and *Copaifera trapezifolia,* Fabaceae) but were not found in their feces. Muriquis defecated on average 10 times a day (R. S. Bueno, unpublished data) but we were not able to define tapirs defecation rate.

We found low similarity (17.2%) in the assemblages of plant species dispersed by muriquis and tapirs, and highly asymmetric complementarity between the two frugivorous. Muriquis complemented tapirs by 79% while tapirs complemented muriquis only with 3%. Only five species of 28 species recorded were dispersed by both tapirs and muriquis (Supplemental Material). The palm *Euterpe edulis* was dispersed exclusively by tapirs, while muriquis were the exclusive dispersers of 23 species.

We conservatively estimated that one muriqui could disperse at least 918 seeds >2 mm per month, or 11,016 seeds per year. One tapir can disperse at least 140 seeds >2 mm per month or 1,680 seeds per year. Therefore, a muriqui troop with 35 individuals is able to disperse at least 385,000 seeds >2 mm/year. In the home range of muriquis studied group (850 ha), 22 individuals of tapirs were identified based on molecular analysis (A. Sanches, unpubl. data), which could disperse 36,960 seeds/year.

### Seed Size Selection

**Figure 3:**
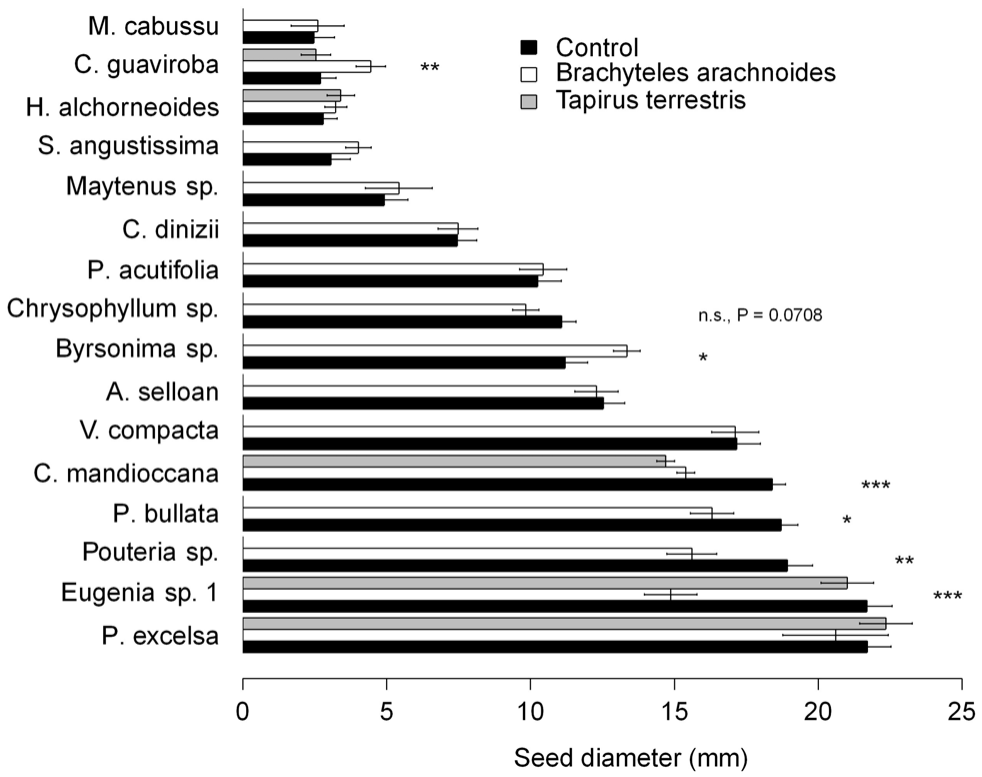
Comparative seed size selection by muriquis (*Brachyteles arachnoides*) and tapirs (*Tapirus terrestris*) *vs*. control (seeds collected from trees) in Carlos Botelho State Park, Atlantic Forest, Brazil (n.s non significant, * P<0.05, ** P<0.01, *** P<0.001).

Muriquis dispersed seeds from a wide range of sizes, from *Miconia cabussu* (Melastomataceae) with less than 2 mm to *Parinari excelsa* (Chrysobalanaceae) with 23.4 mm seed diameter (Figure 3). For tapirs, the smallest dispersed seed was *Hieronyma alchorneoides* (Phyllanthaceae) with 3.1 mm, and the largest was *Eugenia* sp1 with 29.2 mm diameter. In general muriquis selected a subset of relatively small seeds in plant species (*Eugenia* sp., *Pouteria* sp., *P. bullata* and *C. mandioccana*) with seeds over 15 mm of diameter (t >2.4, d.f. = 686, P<0.0114). Exceptions to this pattern were *Parinaria excelsa* and *Vantanea compacta* for which we collected only two and ten seeds, respectively, from muriquis feces. In contrast, in 8 out of 10 species with small seeds (<13 mm of diameter) muriquis did not select for a subset of seed size but in *Byrsonimia* sp. (t = 2.36, d.f. = 686, P = 0.0187) and *C. guaviroba* (t = 2.35, d.f. = 686, P = 0.0197) muriquis selected for a subset of large seeds. Overall tapirs did not seem to select fruit/seed sizes except for *C. mandioccana* for which seeds in tapir dung were on average significantly smaller than the average of the whole set of available seeds (t = 6.69, d.f. = 686, P<0.0001).

Further, from an extended review of the literature on seeds dispersed by muriquis and tapirs in the Atlantic forest (Table S1), we found that muriquis disperse a significantly higher number of seeds from small-seeded species (<15 mm), while tapirs additionally disperse a few large-seeded species (>25 mm) (Figure S1).

### Germination Trial

**Figure 4:**
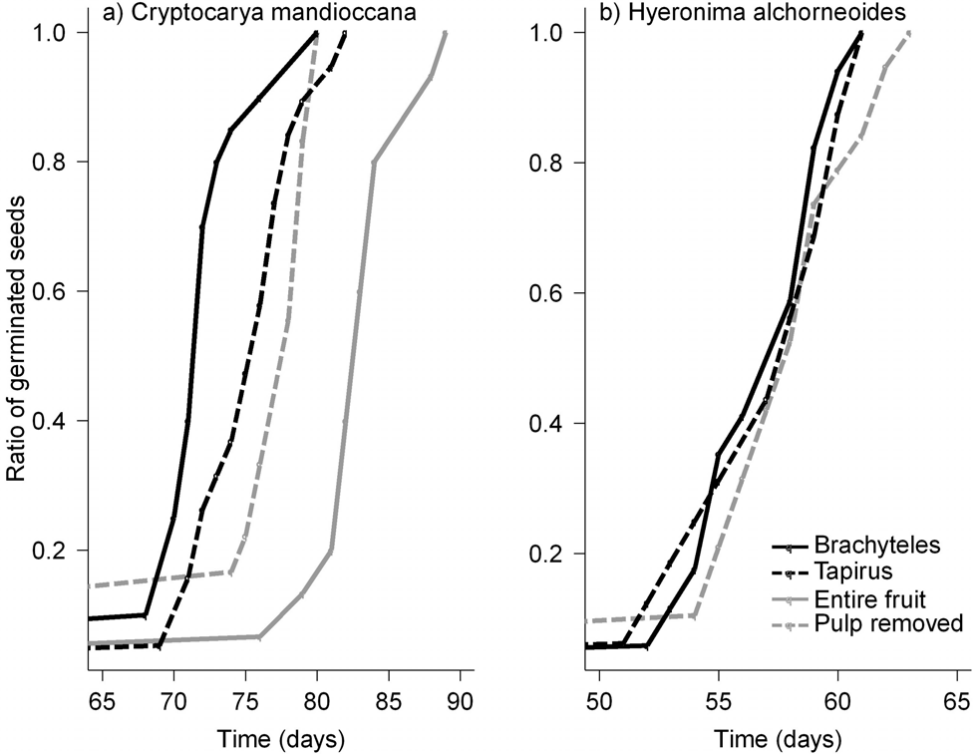
Germination speed of two plant species dispersed by muriquis (*Brachyteles arachnoides*) or tapirs (*Tapirus terrestris*) *vs*. control treatments: *Cryptocarya mandioccana* (a) and *Hieronyma alchorneoides* (b).

Germination time of *C. mandioccana* (Figure 4a) was significantly shortened for seeds that were ingested and defecated by muriquis (z >4.9, P<0.0001) and tapirs (z >4.2, P<0.0001) compared with seeds manually removed from fruits and those that remain within the fruit pulp. Also seeds manually removed from fruits had a shorter germination time compared with seeds embedded in the pulp (z = 2.4, P<0.0001). All these effects were solely due to the fact that ingestion, defecation, and manual extraction of seeds, reduced the germination time of seeds of *C. mandioccana* roughly by 5–8 days, but once seeds started to germinate in any treatment the germination success was similar in all cases. In contrast for *H alchorneoides* no effect on germination was observed when we compared ingested and defecated seeds by muriquis and tapir with seeds manually removed from the pulp (z = 1.4, P = 0.17).

### Spatial Seed Deposition

Out of the 266 registered muriqui defecation events, 55 (21%) feces reached the ground in a cohesive block (<0.3 m), while in 211 cases (79%) feces were fragmented as they hit the understory, generating a locally widespread seed rain, from 0.3 m to more than 3 m radius, especially when on steep slopes. Fifty-nine feces (22%) fell in the water, 26 (10%) on dry valleys, 85 (32%) on slopes, and 90 (34%) on hilltops. We found that for the 49 tapir feces, 11 (22%) were in the water, five (10%) on dry valley, 16 (33%) on slopes and 17 (35%) on hilltops.

For muriquis, the average distance of the defecations from trees with DBH >40 cm was 2.9±1.9 m (range = 0.1–8.4 m, N = 92) and for tapirs 2.5±1.7 m (0.2–9.1 m, N = 49), showing a random pattern with no preference in defecating near tree trunks. On just two occasions, we were able to accurately measure the seed dispersal distance for muriquis and found that *Eugenia* sp. 1 seeds were dispersed 169 and 693 m from the parent tree. On 16 occasions, it was possible to measure the distance of muriqui-defecated seeds from a conspecific adult, with average of 12.7±9.2 m (2–25 m). For tapirs we never identified a conspecific adult inside the 25 m radius circle searched.

**Figure 5:**
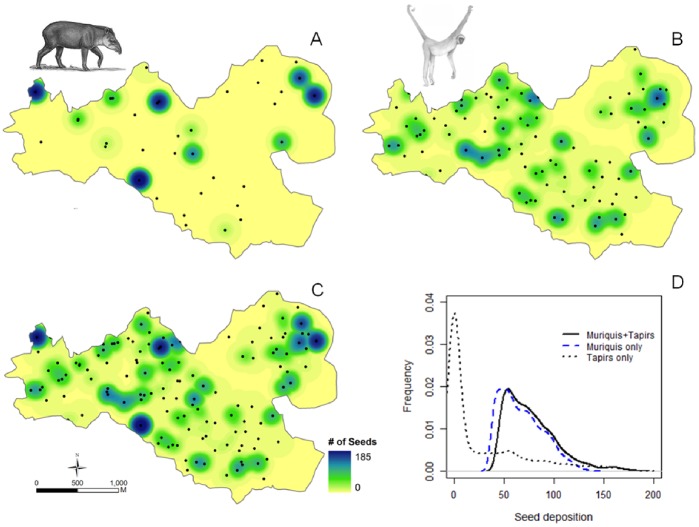
Spatial distribution of dispersed seeds: tapir (*Tapirus terrestris*) (A), muriqui (*Brachyteles arachnoides*) (B) and combined dispersed seeds by both megafrugivores (C) in 850 ha Atlantic forest, Brazil. Frequency of seed deposition at any random position recorded on muriquis and tapirs feces estimated by 1,000 random points overlapped on the maps A, B and C (D).

When analyzing the spatial distribution of feces for muriquis only, tapirs only and muriquis+tapirs, we observed that the spatial distribution of seeds deposited by muriquis was more scattered. The average empty space for muriquis was 122.6±2.7 m, while for tapirs it was almost double (202.1±4.4 m). But if we combine both dispersers the average empty space decreases to 106.6±2.3 m (Figure 5). The t-test was highly significant when comparing muriquis+tapirs *vs.* muriquis only (t = −4.5437, P<<0.001), muriquis+tapirs *vs.* tapirs only (t = −19.12, P<<0.001) and muriquis only *vs.* tapirs only (t = −15.37, P<<0.001). Seed deposition at any random position within study site were estimated as 68.2±0.3 seeds for muriquis only, 28.4±0.6 for tapirs only and 78.5±0.4 for muriquis+tapirs (Figure 5). The t-test was highly significant when comparing muriquis+tapirs *vs.* muriquis only (t = 20.65, P<<0.001), muriquis+tapirs *vs.* tapirs only (t = 71.42, P<<0.001) and muriquis only *vs.* tapirs only (t = 60.62, P<<0.001).

## Discussion

Tapirs and muriquis have distinct seed dispersal effectiveness (both quantitative, qualitative and spatial), and differ in the diversity of plant species, total number of seeds, aggregation and seed sizes dispersed (Table 1). The quantity of seeds and diversity of dispersed species by muriquis was similar to other studies conducted at the same forest type [26], [39], [40]. However, the low diversity and quantity of seeds dispersed by tapirs was not expected, especially considering that about 83% of woody plants in the study site have fleshy fruits that are dispersed by animals [23], [29]. For instance, other studies found that tapirs dispersed more species even where species with fleshy fruits were less abundant [33]. A recent review on tapir seed dispersal shows that the diversity of seeds dispersed by tapirs is much higher in the Amazon, cerrado or semideciduous forests than in ombrophylous forests (our study area) [41].

**Table 1:** Characteristics and seed dispersal effectiveness of tapirs (*Tapirus terrestris*) and muriquis (*Brachyteles arachnoides*) in the Brazilian Atlantic Forest.

Frugivore traits/role	Tapirs	Muriquis
Max. Body mass (kg)	250	12
Local density (ind./km^2^)	2.58*	4.11
Biomass (kg/km^2^)	601.14	49.32
Home range (ha)	470**	850
Number of fruit species eaten	6	31
Number of scats analyzed (N)	49	106
Spatial seed rain	Clumped	Scattered
% feces with seeds	32	87
Number of plant species dispersed	6	28
Maximum number of species per scat	2	5
Number of exclusive species	1	23
Maximum seed width (mm) ofdispersed seeds	29.2	23.4
Maximum seed length (mm) ofdispersed seeds	46.3	30.2
Mean number of seeds >2 mm/scat	72.5±48.8	23.3±17.5
Minimum of seeds >2 mm dispersed/month/individual	140	918
Minimum of seeds >2 mm dispersed/year	1.680	11.016

* A. Sanches, unpublished data;

** [65].

### Seed Size

The seed size (width) is typically employed as the main characteristic that defines the array of dispersers [42]. Since small seeds generally have a wide spectrum of dispersers, resulting in a potential compensation mechanism in the absence of one disperser or another, as the seed size increases, diversity of dispersers decrease [19], [43]. In addition, larger seeds have more nutritional reserve, favoring growth in low light and nutrient availability [44]. The largest seeds found at our study site were *Attalea dubia, Eugenia* sp. 1, *Pouteria* sp., *Pouteria bullata* and *Parinari excelsa,* all with more than 15 mm of average width. All but *Attalea dubia* rely exclusively on tapirs and muriquis for seed dispersal and are heavily predated by squirrels and bruchids. Although other studies report *Attalea* seeds being dispersed by tapirs [45], [46], we found no *Attalea* seed in tapir feces in our study area.

The tapir behavior of defecating in latrines can reduce seed predation by bruchids and rodents [47], and the aggregated spatial distribution of many palm species, including those with large seeds such as *Maximiliana maripa,* is attributed to tapir seed dispersal [48]. In our study area, we do not know any plant species with spatial distribution that could reflect dispersal in tapir’s latrines.

In terms of seed size selection, in general muriquis do not select seeds of small-seeded species (<13 mm), as they ingested the whole range of sizes available in the plant community, while in plant species with seeds larger than 15 mm muriquis selected for a subset of relatively small seeds. The seed size limit that an adult muriqui can ingest is 23 mm, 22% larger than the seed size limit swallowed by woolly monkeys (*Lagothrix lagotricha*) in Colombia [15]. For tapirs, there is no apparent size limit, since they are able to swallow seeds with width up to 40 mm [33], [49], which is much larger than any seed species found at our study site. A literature review of seed sizes dispersed by tapirs and muriquis in the Brazilian Atlantic forest also support our finding from Carlos Botelho State Park (Table S1, Figure S1).

### Spatial Seed Deposition

Seed shadow in rainforests basically consists of the spatial distribution of the seeds generated by animal dispersers, which is mainly influenced by the gut passage time, defecation pattern (frequency), movement and habitat use [50]. In our study area, tapirs have a solitary habit and occur in relatively lower density than muriquis (2.58 individual/km^2^, A. Sanches, unpublished data) and defecate mostly in latrines or in water bodies, while muriquis occur in relatively higher density (4.11 individual/km^2^, R. S. Bueno, unpublished data), have a fission-fusion strategy with sub-groups consisting of about 5 to 20 or more individuals, which means a higher probability of seed dispersal across different microsites. So, while tapirs clearly generate a clumped distribution of seeds, muriquis generate both scattered and clumped seed dispersal, a pattern also reported for *Ateles paniscus* in Peru [51]. Indeed, the muriquis scattered seeds throughout their home range along diurnal travel routes by defecating on average 10 times a day (R. S. Bueno, unpublished data), but also because most of the feces hit the leaves and branches of the understory, reaching the ground often spread around 3 meters. This in-transit seed deposition alternates with clumped seed deposition due to the concentration of seeds under muriquis’ sleeping sites. However, the muriquis in our study used 23 different sleeping sites and rarely used the same sleeping site for two consecutive days, which can mitigate this clumped distribution. This difference of defecation pattern between muriquis and tapirs is reflected in our analysis for spatial distribution of feces and seed deposition where muriquis left half the empty space when compared to tapirs. Therefore, the probability of a seed dispersed by tapirs of colonizing a favorable site is much less than that of seeds dispersed by muriquis.

### Seed Germination

We found no difference in the germination success of seeds that were dispersed by tapirs and muriquis. The gut treatment of these frugivores shortened the germination times of *C. mandioccana*, indicating that pulp removal is relevant for germination in this species, and probably also in other species dispersed by muriquis [26], [40]. Ingestion and defecation reduced the germination time of *C. mandioccana* by 5 to 8 days compared with seeds manually removed from the pulp and seeds remaining in the pulp. Although this may seem a small improvement in germination for *C. mandioccana*, it can become quite relevant if seed predation and decay are minimized via shorter exposure to seed predators and microbes, and also via removal of pulp that may act as an incubator for fungus and bacteria likely to rot seed tissues. This may be particularly relevant if the main demographic sieve controlling *C. mandioccana* population is at the seed stage. Rodents and invertebrates prey heavily upon *C. mandioccana* seeds, particularly beneath the parent tree (L. Culot, unpublished data).

### Redundancies and Complementarities

Muriquis and tapirs are the last representatives of a formerly rich neotropical frugivore megafauna [5], [52]. While muriquis disperse a higher diversity and quantity of seeds, spreading them throughout the forest, there is a seed size limit that they disperse, they avoid secondary forests and they do not cross large open areas [53], [54]. Tapirs show no seed size restriction (for the local flora), use degraded forest and cross open areas, including going outside the park limit. Thus, despite dispersing a lower diversity of species and depositing them in a clumped pattern, they provide an important function in seed dispersal that is not performed by muriquis. Tapirs visit frequently the fragments neighboring the park and may move seeds to these areas (R. Bueno unpublished data).

Although we did not evaluate post-dispersal seed fate, muriqui feces attract higher abundance and diversity of secondary seed dispersers, such as dung beetles, than tapir feces (L. Culot, M. Boutefeu and E. Bovy unpublished data), increasing the probability of germination and escape from seed predation. On the other hand, some authors have found that post-dispersal seed predation on seeds in tapir dung is lower than seeds not immersed in latrines [55].

We found that muriquis dispersed 28 species during 18 mo of this study, but it is likely they eat a much higher diversity of fruits. Studies on muriqui diet in neighboring sites recorded another 36 fruit species that were not observed in muriquis feces in our study but are known to occur in our study area [56], [57]. In addition, 84% of the 434 tree species in our study area have fleshy fruits [23]. Tapirs, on the other hand, are less frugivorous in the ombrophilous Atlantic forest than in the Amazon and semideciduous forests and cerrados [58]. Although, we are aware that our sample size represents a specific period of forest phenology and fruit availability, we believe that the results indicate a general pattern for both species in the Atlantic forest (see Table S2). During periods of fruit scarcity muriquis rely mainly on leaves [31], but our data showed that even when fruits are plenty, tapirs have mainly a folivorous diet, as we saw many uneaten fleshy fruits on the forest floor.

Large-bodied primates and ungulates are threatened by habitat loss and poaching all over the Neotropics [59]. Both muriquis and tapirs have few remaining populations in the remaining 12% of the Brazilian Atlantic forest [21], [60]. Both fragmented and continuous defaunated forests are already suffering important cascading trophic effects. For instance, the extinction of muriquis will cause a profound change in seed rain and will increase dispersal limitation by many plant species. Moreover, the extinction of both muriquis and tapirs will affect the seed dispersal of large-seeded species (e.g. Sapotaceae, Chrysobalanaceae and Myrtaceae) increasing extinction risk for these plant species. We compiled 302 plant species dispersed by tapirs or muriquis in the Brazilian Atlantic forest (Table S2).

The extinction of tapirs may also disrupt long-distance dispersal and colonization of large seeded species into open areas or fragments [61]. Other large primates (e.g. howler monkeys *Alouatta* spp.) and ungulates (peccaries and deer) do not replace the seed dispersal services by muriquis and tapirs. Howlers are highly folivorous, have a much smaller home range and defecate mostly beneath the sleeping trees [16], [62], while peccaries and deer are primarily seed predators [63], [64].

Therefore, the extinction of these last megafrugivores may translate into a series of impoverished plant-animal interactions, whereby the persistence of many plant species will have to rely on human interventions. Our study demonstrates that both megafrugivores are essential for promoting distinct seed dispersal services in tropical forests.

## Supporting Information

Figure S1 **Comparative of seed size (diameter) of plant species eaten by muriquis (** ***Brachyteles arachnoides*** **and** ***B*** **.** ***hypoxanthus*** **) and tapirs (** ***Tapirus terrestris*** ) in the Atlantic forest (*P<0.05, ** P<0.01, *** P<0.001, ns = not significant).
(TIF)

Table S1 **Species dispersed and number of seeds per scat by tapirs (** ***Tapirus terrestris*** **) and muriquis (** ***Brachyteles arachnoides*** **) in Carlos Botelho State Park, Atlantic Forest, Brazil.**
(DOC)

Table S2 **Compiled information from a literature review of seed size of species dispersed by muriquis (** ***Brachyteles arachnoides*** **and** ***hypoxanthus*** **) and tapirs (** ***Tapirus terrestris*** **) in the Brazilian Atlantic forest.** Plant names of eaten fleshy fruits follow [33].
(DOC)
