# Supplementary figures and images for: Functional Redundancy and Complementarities of Seed Dispersal by the Last Neotropical Megafrugivores
Source: PLoS One. 2013 Feb 7;8(2):e56252. doi: 10.1371/journal.pone.0056252 (PMC3567037; doi:10.1371/journal.pone.0056252)

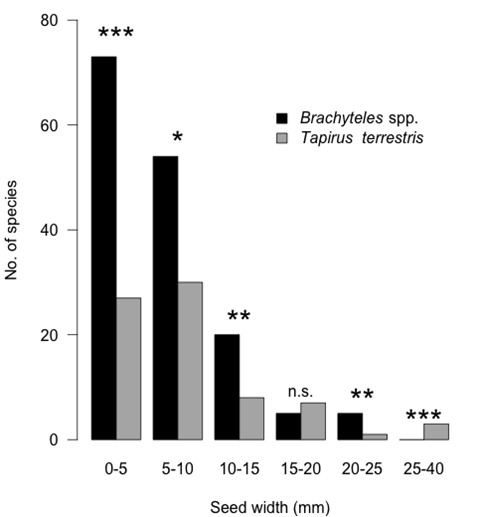

Supplement: Figure S1 — Comparative of seed size (diameter) of plant species eaten by muriquis ( Brachyteles arachnoides and B . hypoxanthus ) and tapirs ( Tapirus terrestris ) in the Atlantic forest (*P<0.05, ** P<0.01, *** P<0.001, ns = not significant). (TIF) [file pone.0056252.s001.tif]
