# Supplementary material for: Functional Redundancy and Complementarities of Seed Dispersal by the Last Neotropical Megafrugivores
Source: PLoS One. 2013 Feb 7;8(2):e56252. doi: 10.1371/journal.pone.0056252 (PMC3567037; doi:10.1371/journal.pone.0056252)
Supplement: Table S1 — Species dispersed and number of seeds per scat by tapirs ( Tapirus terrestris ) and muriquis ( Brachyteles arachnoides ) in Carlos Botelho State Park, Atlantic Forest, Brazil. (DOC) [file pone.0056252.s002.doc]

| **Family** | **Species** | **Average (SD) seeds/defecation (N)(Muriquis)** | **Average (SD) seeds/defecation (N) (Tapir)** | **Average seeds per fruit** |
| --- | --- | --- | --- | --- |
|
| Araliaceae | *Schefflera angustissima* | 18.00 ± 3.19 (16) |  | 1 |
| Arecaceae | *Euterpe edulis* |  | 13.7 ± 7.5 (3) | 1 |
| Boraginaceae | *Cordia* sp*.* | 4.83 ± 1.24 (6) |  | 1 |
| Canellaceae | *Cinnamodendron dinizii* | 13.45 ± 2.10 (11) |  | 3 |
| Celastraceae | *Maytenus* sp*.* | 9.0 ± 1.4 (11) |  | 1 |
| Chrysobalanaceae | *Parinari excelsa* | 2 (2) | 9 (1) | 1 |
| Euphorbiaceae | *Alchornea triplinervia* | 11.00 ± 3.08 (4) |  | 3 |
| Humiriaceae | *Vantanea compacta* | 3.33 ± 0.66 (3) |  | 1 |
| Lauraceae | *Cryptocarya mandioccana* | 8.25 ± 0.83 (20) | 46.2 ± 13.22 (3) | 1 |
| Malphighiaceae | *Byrsonima* sp*.* | 6.83 ± 1.01 (6) |  | 1 |
| Melastomataceae | *Miconia cabussu* | 10.57 ± 2.03 (7) |  | 5 |
| Menispermaceae | *Abuta selloana* | 5.13 ± 0.69 (8) |  | 1 |
| Myrtaceae | *Campomanesia guaviroba* | 15.82 ± 2.42 (17) | 86.5 ± 15.5 (6) | 5 |
| Myrtaceae | *Eugenia* sp. 1 | 3.50 ± 0.64 (4) | 20.4 ± 3.6 (3) | 1 |
| Myrtaceae | *Eugenia* sp. 2 | 2.25 ± 0.47 (4) |  | 1 |
| Myrtaceae | Myrtaceae unidentified | 3.26 ± 1.1 (9) |  | 1 |
| Phyllanthaceae | *Hieronyma alchorneoides* | 19.75 ± 2.55 (20) | 100.6 ± 43.0 (4) | 1 |
| Rubiaceae | *Posoqueria acutifolia* | 5.57 ± 0.89 (7) |  | 6 |
| Rubiaceae | *Psychotria mapoureoides* | 12.25 ± 2.62 (4) |  | 4 |
| Sapotaceae | *Chrysophyllum* sp*.* | 7.57 ± 1.04 (7) |  | 2 |
| Sapotaceae | *Chrysophyllum viride* | 2.8 ± 0.37 (5) |  | 3 |
| Sapotaceae | *Micropholis gardneriana* | 5.5 ± 0.86 (4) |  | 1 |
| Sapotaceae | *Pouteria bullata* | 2.00 ± 0.44 (6) |  | 2 |
| Sapotaceae | *Pouteria* sp*.* | 2.25 ± 0.25 (4) |  | 2 |
| Solanaceae | *Solanum pseudo-quina* | 19.43 ± 3.27 (7) |  | 10 |
| Symplocaceae | *Symplocos* sp*.* | 4.9 ± 2.3 (4) |  | 1 |
| Urticaceae | *Coussapoa microcarpa* | > 150 (12) |  | >20 |
| n ident 1 | *n ident 1* | 8.5 ± 1.5 (6) |  |  |
| n ident 2 | *n ident 2* | 5.56 ± 1.6 (2) |  |  |
| n ident 3 | *n ident 3* | 3(1) |  |  |
| n ident 4 | *n ident 4* | 2(1) |  |  |
