# Supplementary material for: Functional Redundancy and Complementarities of Seed Dispersal by the Last Neotropical Megafrugivores
Source: PLoS One. 2013 Feb 7;8(2):e56252. doi: 10.1371/journal.pone.0056252 (PMC3567037; doi:10.1371/journal.pone.0056252)
Supplement: Table S2 — Compiled information from a literature review of seed size of species dispersed by muriquis ( Brachyteles arachnoides and hypoxanthus ) and tapirs ( Tapirus terrestris ) in the Brazilian Atlantic forest. Plant names of eaten fleshy fruits follow [33]. (DOC) [file pone.0056252.s003.doc]

| **Family** | **Species** | **Frugivore Species** | **Mean seed width (mm)** | **Reference** |
| --- | --- | --- | --- | --- |
| Acanthaceae | *Mendoncia coccinea* | *Brachyteles arachnoides* | 5 | [1] |
| Achariaceae | *Carpotroche brasiliensis* | *Brachyteles arachnoides, B. hypoxanthus* | 13 | [1], [2] |
| Amaranthaceae | *Chamissoa* sp*.* | *Brachyteles arachnoides* | 7 | [1] |
| Anacardiaceae | *Spondias dulcis* | *Brachyteles hypoxanthus* |  | [2] |
| Anacardiaceae | *Spondias mombin* | *Tapirus terrestris* | 20 | [3], [4], [5], [6], [7], [8], [9] |
| Anacardiaceae | *Spondias* sp*.* | *Tapirus terrestris* | 20 | [10] |
| Anacardiaceae | *Tapirira guianensis* | *Tapirus terrestris* | 9 | [3], [4] |
| Anacardiaceae | *Tapirira marchandii* | *Brachyteles arachnoides* |  | [1] |
| Anacardiaceae | *Thyrsodium schomburgkianum* | *Tapirus terrestris* | 10 | [3] |
| Anarcadiaceae | *Tapirira guianensis* | *Brachyteles arachnoides, B. hypoxanthus* | 5 | [1], [11], [12], [13] |
| Annonaceae | *Annona cacans* | *Tapirus terrestris* | 10 | [14] |
| Annonaceae | *Annona cacans* | *Brachyteles hypoxanthus* | 10 | [13] |
| Annonaceae | *Annona crassiflora* | *Tapirus terrestris* | 10 | [3] |
| Annonaceae | *Annona muricata* | *Tapirus terrestris* | 10 | [3] |
| Annonaceae | *Annona* sp. | *Brachyteles arachnoides* | 8 | [12] |
| Annonaceae | *Annona* sp. | *Tapirus terrestris* | 8 | [8], [9], [15] |
| Annonaceae | *Duguetia lanceolata* | *Brachyteles arachnoides* | 5 | [16], [17] |
| Annonaceae | *Guatteria australis* | *Brachyteles arachnoides, B. hypoxanthus* | 5 | [13], [18] |
| Annonaceae | *Guatteria odontopetala* | *Brachyteles arachnoides* | 5 | [1] |
| Annonaceae | *Guatteria pogonopus* | *Brachyteles hypoxanthus* | 5 | [13] |
| Annonaceae | *Guatteria* sp*.* | *Brachyteles arachnoides* |  | [19] |
| Annonaceae | *Rollinia laurifolia* | *Brachyteles hypoxanthus* |  | [13] |
| Annonaceae | *Rollinia sericea* | *Brachyteles arachnoides* | 4 | [20] |
| Annonaceae | *Rollinia* sp*.* | *Brachyteles arachnoides* | 9.3 | [1] |
| Annonaceae | *Rollinia* sp*.* | *Tapirus terrestris* | 5 | [9], [15] |
| Aquifoliacae | *Ilex* sp. | *Tapirus terrestris* | 3 | [14] |
| Aquifoliaceae | *Ilex paraguaiensis* | *Tapirus terrestris* | 3 | [14] |
| Araceae | *Anthurium harrisii* | *Brachyteles arachnoides* |  | [20] |
| Araceae | *Heteropsis oblongfolia* | *Brachyteles arachnoides* |  | [20] |
| Araceae | *Monstera adansonii* | *Brachyteles arachnoides* |  | [20] |
| Araceae | *Philodendron appendiculattum* | *Brachyteles arachnoides* | 0.5 | [20] |
| Araceae | *Philodendron corcovadense* | *Brachyteles arachnoides* | 2 | [20] |
| Araceae | *Phylodendron imbe* | *Brachyteles arachnoides* |  | [18] |
| Araliaceae | *Schefflera calva* | *Brachyteles arachnoides* | 8 | [12] |
| Arecaceae | *Acrocomia aculeata* | *Tapirus terrestris* | 20.01 | [14] |
| Arecaceae | *Butia capitata* | *Tapirus terrestris* | 10 | [21] |
| Arecaceae | *Elaeis guineensis* | *Tapirus terrestris* | 20 | [3] |
| Arecaceae | *Euterpe edulis* | *Tapirus terrestris* | 11 | [3], [22] |
| Arecaceae | *Syagrus oleracea* | *Tapirus terrestris* | 27 | [22] |
| Arecaceae | *Syagrus romanzoffiana* | *Tapirus terrestris* | 16 | [15], [22], [23], [24] |
| Arecaceae | *Syagrus romanzoffiana* | *Brachyteles arachnoides* | 16 | [16] |
| Boraginaceae | *Cordia sellowiana* | *Brachyteles arachnoides* | 7 | [16], [25] |
| Boraginaceae | *Cordia* sp*.* | *Tapirus terrestris* | 4 | [7], [14], [26] |
| Boraginaceae | *Cordia* sp*.* | *Brachyteles arachnoides* | 4 | [26] |
| Boraginaceae | *Cordia* sp*.* 2 | *Brachyteles arachnoides* | 4 | [1] |
| Bromeliaceae | *Bromelia balansae* | *Tapirus terrestris* | 5.43 | [14] |
| Burseraceae | *Protium heptaphyllum* | *Tapirus terrestris* | 7.5 | [3] |
| Burseraceae | *Protium heptaphyllum* | *Brachyteles arachnoides* | 13.5 | [1] |
| Burseraceae | *Protium widgrenii* | *Brachyteles arachnoides* | 9 | M Galetti |
| Burseraceae | *Protium* sp*.* | *Tapirus terrestris* | 7.5 | [9] |
| Cactaceae | *Rhipsalis* sp*.* | *Brachyteles arachnoides* | 0.5 | [1] |
| Cactaceae | *Rhipsalis* sp*.* 1 | *Brachyteles arachnoides* | 0.5 | [11] |
| Cactaceae | *Pereskia aculeata* | *Brachyteles arachnoides* | 1 | M Galetti |
| Canellaceae | *Cinnamodendron dinisii* | *Brachyteles arachnoides* | 3 | [12], [18], [20], [26], [27] |
| Cannabaceae | *Celtis* sp*.* 1 | *Brachyteles hypoxanthus* |  | [2] |
| Cannabaceae | *Celtis* sp*.* 2 | *Brachyteles hypoxanthus* |  | [2] |
| Cannabaceae | *Celtis* sp*.* 3 | *Brachyteles hypoxanthus* |  | [2] |
| Cannabaceae | *Celtis spinosa* | *Brachyteles arachnoides* |  | [16], [25] |
| Caricaceae | *Jacaratia spinosa* | *Brachyteles arachnoides* | 5 | [16], [25], [27] |
| Caricaceae | *Jacaratia spinosa* | *Tapirus terrestris* | 5 | [4], [7], [14] |
| Celastraceae | *Cheiloclinium serratum* | *Brachyteles arachnoides* | 21 | [18][27] |
| Celastraceae | *Maytenus alanternoides* | *Brachyteles arachnoides* | 9 | [11], [12] |
| Celastraceae | *Maytenus aquifolium* | *Brachyteles arachnoides* | 4 | [20] |
| Celastraceae | *Maytenus cestrifolia* | *Brachyteles arachnoides* | 6 | [18] |
| Celastraceae | *Maytenus* sp*.* | *Brachyteles hypoxanthus* | 6 | [13] |
| Celastraceae | *Maytenus* sp*.* | *Tapirus terrestris* | 7 | [8], [26] |
| Celastraceae | *Salacia eliptica* | *Brachyteles arachnoides* | 13.6 | [20] |
| Chrysobalanaceae | *Licania khunthiana* | *Brachyteles hypoxanthus* | 8 | [2], [13] |
| Chrysobalanaceae | *Licania* sp*.* | *Brachyteles arachnoides* | 8 | [1] |
| Chrysobalanaceae | *Licania* sp*.* | *Tapirus terrestris* | 8 | [8] |
| Chrysobalanaceae | *Parinari excelsa* | *Brachyteles arachnoides* | 16 | [20], [26] |
| Chrysobalanaceae | *Parinari excelsa* | *Tapirus terrestris* | 16 | [5], [7], [26] |
| Clusiaceae | *Clusia* sp*.* 1 | *Tapirus terrestris* | 2 | [4], [9] |
| Clusiaceae | *Clusia* sp. 2 | *Brachyteles hypoxanthus* | 2 | [2] |
| Clusiaceae | *Clusia* sp*.* 3 | *Brachyteles hypoxanthus* | 2 | [2] |
| Clusiaceae | *Clusia* sp*.* 4 | *Brachyteles hypoxanthus* | 2 | [2] |
| Clusiaceae | *Garcinia brasiliensis* | *Tapirus terrestris* | 10.34 | [28] |
| Clusiaceae | *Garcinia brasiliensis* | *Brachyteles hypoxanthus* | 10.34 | [13] |
| Clusiaceae | *Garcinia gardneriana* | *Tapirus terrestris* | 14 | [28] |
| Clusiaceae | *Garcinia gardneriana* | *Brachyteles arachnoides* | 14 | [20] |
| Clusiaceae | *Tovomitopsis* sp*.* | *Brachyteles arachnoides* |  | [1] |
| Combretaceae | *Buchenavia kleinii* | *Brachyteles arachnoides* | 8 | [18] |
| Combretaceae | *Buchenavia* sp*.* | *Brachyteles hypoxanthus* | 8 | [13] |
| Elaeocarpaceae | *Sloanea monosperma* | *Brachyteles arachnoides* | 7 | [16] |
| Erythroxylaceae | *Erythroxylum* sp*.* | *Tapirus terrestris* | 5 | [3] |
| Euphorbiaceae | *Alchornea glandulosa* | *Brachyteles hypoxanthus* | 5 | [13] |
| Euphorbiaceae | *Alchornea triplinervia* | *Brachyteles arachnoides, B. hypoxanthus* | 4 | [12], [13], [26] |
| Euphorbiaceae | *Pera obovata* | *Brachyteles arachnoides* | 0.3 | [11] |
| Euphorbiaceae | *Sapium glandulosum* | *Brachyteles arachnoides* | 0.3 | [11] |
| Fabaceae | *Acacia grandistipula* | *Brachyteles arachnoides* | 7 | [11], [12] |
| Fabaceae | *Copaifera langsdorffii* | *Tapirus terrestrisus* | 9 | [22] |
| Fabaceae | *Copaifera langsdorfii* | *Brachyteles arachnoides* | 9 | [11], [17] |
| Fabaceae | *Copaifera trapezifolia* | *Brachyteles arachnoides* | 8 | [11], [12], [20] |
| Fabaceae | *Enterolobium contortisiliquum* | *Tapirus terrestris* | 9.17 | [14], [22] |
| Fabaceae | *Hymenaea altissima* | *Brachyteles arachnoides* | 20 | [20] |
| Fabaceae | *Hymenaea courbaril* | *Tapirus terrestris* | 20 | [14] |
| Fabaceae | *Hymenaea courbaril* | *Brachyteles arachnoides* | 20 | [16], [17], [18], [27] |
| Fabaceae | *Hymenaea oblongifolia* | *Tapirus terrestris* | 20 | [9] |
| Fabaceae | *Inga fagifolia* | *Brachyteles arachnoides* | 9 | [11], [12] |
| Fabaceae | *Inga marginata* | *Brachyteles arachnoides* | 10 | [18] |
| Fabaceae | *Inga sessilis* | *Brachyteles arachnoides* | 10 | [11], [18], [19] |
| Fabaceae | *Inga* sp*.* | *Tapirus terrestris* | 9 | [10], [22] |
| Fabaceae | *Inga striata* | *Brachyteles arachnoides* | 6 | [16], [31] |
| Fabaceae | *Swartzia* sp*.* | *Brachyteles hypoxanthus* | 10 | [2] |
| Fabaceae | *Swartzia* sp*.* | *Tapirus terrestris* | 10 | [6] |
| Fabaceae | *Zollernia ilicifolia* | *Brachyteles arachnoides* |  | [11], [12] |
| Humiriaceae | *Vantanea compacta* | *Brachyteles arachnoides* | 23 | [11], [26] |
| Lauraceae | *Beilschmiedia emarginata* | *Brachyteles arachnoides* |  | [18] |
| Lauraceae | *Cinnamomum glaziovii* | *Brachyteles arachnoides* | 5 | [19] |
| Lauraceae | *Cinnamomum* sp*.* | *Brachyteles arachnoides* |  | [11] |
| Lauraceae | *Cryptocarya archersoniana* | *Brachyteles arachnoides* | 22 | [11], [20] |
| Lauraceae | *Cryptocarya mandioccana* | *Tapirus terrestris* | 15 | [26] |
| Lauraceae | *Cryptocarya mandioccana* | *Brachyteles arachnoides, B. hypoxanthus* | 15 | [12], [20], [26] |
| Lauraceae | *Endlicheria glomerata* | *Brachyteles hypoxanthus* | 13 | [2] |
| Lauraceae | *Endlicheria glomerata* | *Brachyteles arachnoides* | 13 | [2] |
| Lauraceae | *Nectandra* sp*.* | *Brachyteles arachnoides* |  | [27] |
| Lauraceae | *Ocotea spixiana* | *Brachyteles arachnoides* | 5 | [17] |
| Lauraceae | *Ocotea catharinensis* | *Brachyteles arachnoides* | 5 | [11], [12], [19] |
| Lauraceae | *Ocotea corymbosa* | *Brachyteles arachnoides* | 5 | [11][13] |
| Lauraceae | *Ocotea diospyrifolia* | *Brachyteles arachnoides* | 10 | [18] |
| Lauraceae | *Ocotea glaziovii* | *Brachyteles arachnoides* |  | [11], [12] |
| Lauraceae | *Ocotea insignis* | *Brachyteles hypoxanthus* |  | [2][13] |
| Lauraceae | *Ocotea kuhlmannii* | *Brachyteles arachnoides* |  | [11], [12] |
| Lauraceae | *Ocotea odorifera* | *Brachyteles arachnoides* | 5 | [18] |
| Lauraceae | *Ocotea porosa* | *Brachyteles arachnoides* | 13 | [18] |
| Lauraceae | *Ocotea pulchella* | *Brachyteles arachnoides* | 4 | [11], [12] |
| Lauraceae | *Ocotea silvestris* | *Brachyteles arachnoides* |  | [11] |
| Lauraceae | *Ocotea* sp*.* | *Brachyteles arachnoides* |  | [1], [18] |
| Lauraceae | *Ocotea* sp. 1 | *Brachyteles hypoxanthus* |  | [2] |
| Lauraceae | *Ocotea* sp. 2 | *Brachyteles hypoxanthus* |  | [2] |
| Lauraceae | *Phyllostemonodaphne geminiflora* | *Brachyteles hypoxanthus* |  | [2], [13] |
| Lauraceae | *Urbanodendron verrucosum* | *Brachyteles hypoxanthus* |  | [2], [13] |
| Lecythidaceae | *Lecythis* sp*.* | *Tapirus terrestris* | 26 | [4] |
| Loganiaceae | *Strychnos* sp*.* | *Tapirus terrestris* | 15 | [7] |
| Loganiaceae | *Strychnos trinervis* | *Brachyteles arachnoides* | 15 | [11] |
| Loganiaceae | *Strychnos triplinervia* | *Brachyteles arachnoides* | 15 | [12] |
| Malphighiaceae | *Byrsonima coriacea* | *Brachyteles arachnoides* | 7 | [1] |
| Malphighiaceae | *Byrsonima ligustrifolia* | *Brachyteles arachnoides* | 7 | [11] |
| Malphighiaceae | *Byrsonima* sp*.* | *Brachyteles arachnoides* | 7 | [26] |
| Malpighiaceae | *Byrsonima* sp*.* | *Tapirus terrestris* | 6.3 | [3], [4], [9], [26] |
| Malpighiaceae | *Byrsonima stipulaceae* | *Brachyteles hypoxanthus* | 10 | [2] |
| Marcgraviaceae | *Marcgravia polyantha* | *Brachyteles arachnoides* | 0.5 | [20] |
| Marcgraviaceae | *Norantea* sp*.* | *Brachyteles arachnoides* | 0.5 | [20] |
| Melastomataceae | *Clidemia debilis* | *Brachyteles hypoxanthus* | 0.5 | [2] |
| Melastomataceae | *Miconia cabussu* | *Brachyteles arachnoides* | 0.5 | [26] |
| Melastomataceae | *Miconia cinnamomifolia* | *Brachyteles arachnoides* | 1 | [19] |
| Melastomataceae | *Miconia dodecandra* | *Brachyteles hypoxanthus* | 0.5 | [13] |
| Melastomataceae | *Miconia* sp*.* | *Brachyteles hypoxanthus* | 0.5 | [2] |
| Melastomataceae | *Miconia* sp*.* | *Tapirus terrestris* | 0.5 | [9] |
| Melastomataceae | *Mouriri glazioviana* | *Brachyteles arachnoides* | 6.5 | Galetti pers obs |
| Melastomataceae | *Mouriri* sp*.* | *Brachyteles arachnoides* | 6.5 | [31] |
| Meliaceae | *Cabralea canjerana* | *Brachyteles arachnoides* | 5 | [11] |
| Menispermaceae | *Abuta selloana* | *Brachyteles arachnoides* | 11 | [18], [26], [27], |
| Monimiaceae | *Mollinedia* cf. *uleana* | *Brachyteles arachnoides* | 9 | [11] |
| Moraceae | *Artocarpus heterophyllus* | *Tapirus terrestris* | 10 | [3] |
| Moraceae | *Clarisia ilicifolia* | *Brachyteles hypoxanthus* | 8.4 | [2] |
| Moraceae | *Ficus enormis* | *Brachyteles arachnoides* | 1.2 | [11] |
| Moraceae | *Ficus gomelleira* | *Brachyteles arachnoides* | 0.5 | [20] |
| Moraceae | *Ficus insipida* | *Brachyteles arachnoides, B. hypoxanthus* | 1 | [13], [18], [20], [27] |
| Moraceae | *Ficus insipida* | *Tapirus terrestris* | 0.5 | [8] |
| Moraceae | *Ficus organensis* | *Brachyteles arachnoides, B. hypoxanthus* | 0.5 | [13], [18], [27] |
| Moraceae | *Ficus* sp*.* | *Tapirus terrestris* | 0.5 | [4], [5], [6], [7], [9], [14], [22] |
| Moraceae | *Ficus* sp*.* | *Brachyteles arachnoides* | 0.5 | [11], [12], [31] |
| Moraceae | *Helicostylis tomentosa* | *Brachyteles hypoxanthus* | 8 | [13] |
| Moraceae | *Helicostylis tomentosa* | *Tapirus terrestris* | 8 | [4],[5], [7], [9] |
| Moraceae | *Sorocea bonplandii* | *Brachyteles arachnoides* | 5 | [18] |
| Musaceae | *Musa dasycarpa* | *Brachyteles arachnoides* | 1 | [20] |
| Myristicaceae | *Virola bicuhyba* | *Brachyteles arachnoides* | 15 | [18], [20] |
| Myristicaceae | *Virola bicuhyba* | *Brachyteles hypoxanthus* | 15 | [2] |
| Myristicaceae | *Virola gardneri* | *Brachyteles arachnoides* | 24 | [20] |
| Myristicaceae | *Virola gardneri* | *Brachyteles hypoxanthus* | 24 | [2] |
| Myrtaceae | *Campomanesia adamantium* | *Brachyteles arachnoides* | 7 | [11], [12] |
| Myrtaceae | *Campomanesia guaviroba* | *Tapirus terrestris* | 6 | [26] |
| Myrtaceae | *Campomanesia guaviroba* | *Brachyteles arachnoides* | 6 | [12], [18], [26], [27] |
| Myrtaceae | *Campomanesia guazumifolia* | *Brachyteles arachnoides* | 5 | [27] |
| Myrtaceae | *Campomanesia phaea* | *Tapirus terrestris* | 7 | Galetti pers obs |
| Myrtaceae | *Campomanesia* sp*.* | *Brachyteles arachnoides* | 7 | [16] |
| Myrtaceae | *Campomanesia xanthocarpa* | *Brachyteles arachnoides* | 4 | [11], [12] |
| Myrtaceae | *Eugenia* aff*. dodonaefolia* | *Brachyteles arachnoides* |  | [11] |
| Myrtaceae | *Eugenia burkartiana* | *Brachyteles arachnoides* |  | [18] |
| Myrtaceae | *Eugenia dodoneaefolia* | *Brachyteles arachnoides* |  | [12] |
| Myrtaceae | *Eugenia involucrata* | *Brachyteles arachnoides* | 7 | [11], [12] |
| Myrtaceae | *Eugenia ligustrina* | *Brachyteles arachnoides* |  | [16], [25] |
| Myrtaceae | *Eugenia pruinosa* | *Brachyteles arachnoides* |  | [18] |
| Myrtaceae | *Eugenia pyriformis* | *Brachyteles arachnoides* | 15 | [16], [25] |
| Myrtaceae | *Eugenia* sp. 1 | *Tapirus terrestris* | 15 | [26] |
| Myrtaceae | *Eugenia* sp. 2 | *Brachyteles arachnoides* |  | [12], [18], [32] |
| Myrtaceae | *Eugenia* sp. 3 | *Brachyteles arachnoides* |  | [11], [18] |
| Myrtaceae | *Eugenia* sp. 4 | *Brachyteles arachnoides* |  | [11], [18] |
| Myrtaceae | *Eugenia* sp. 5 | *Brachyteles arachnoides* |  | [18] |
| Myrtaceae | *Eugenia* sp*.* 6 | *Brachyteles arachnoides, B. hypoxanthus* | | [1], [12], [16], [18], [25], [32] |
| Myrtaceae | *Eugenia stictosepala* | *Brachyteles arachnoides* | 16 | [20] |
| Myrtaceae | *Gomidesia riedeliana* | *Brachyteles arachnoides* | 9 | [12][11] |
| Myrtaceae | *Gomidesia* sp. | *Tapirus terrestris* | 10 | [14] |
| Myrtaceae | *Gomidesia* sp. | *Brachyteles arachnoides* |  | [11], [12] |
| Myrtaceae | *Mangifera indica* | *Tapirus terrestris* | 40 | [22] |
| Myrtaceae | *Marlierea eugeniopsoides* | *Brachyteles arachnoides* | 13 | [11], [12] |
| Myrtaceae | *Marlierea pubipetala* | *Brachyteles arachnoides* |  | [11] |
| Myrtaceae | *Marlierea* sp. 1 | *Brachyteles arachnoides* |  | [11], [18] |
| Myrtaceae | *Marlierea* sp*.* 2 | *Brachyteles arachnoides* |  | [11] |
| Myrtaceae | *Marlierea* sp*.* 3 | *Brachyteles arachnoides* |  | [11] |
| Myrtaceae | *Marlierea suaveolens* | *Brachyteles arachnoides* | 10 | [11], [12] |
| Myrtaceae | *Myrceugenia myrcioides* | *Brachyteles arachnoides* | 8 | [18] |
| Myrtaceae | *Myrcia anacardiifolia* | *Brachyteles arachnoides* | 9 | [18] |
| Myrtaceae | *Myrcia heringii* | *Brachyteles arachnoides* | 1 | [11] |
| Myrtaceae | *Myrcia macrocarpa* | *Brachyteles arachnoides* | 1 | [18] |
| Myrtaceae | *Myrcia pubipetala* | *Brachyteles arachnoides* | 1 | [18], [27] |
| Myrtaceae | *Myrcia pubipetala* | *Brachyteles arachnoides* | 1 | [18] |
| Myrtaceae | *Myrcia* sp*.*1 | *Tapirus terrestris* | 1 | [14] |
| Myrtaceae | *Myrcia* sp*.*2 | *Brachyteles arachnoides, B. hypoxanthus* | 1 | [1], [2], [11], [13] |
| Myrtaceae | *Myrcia* sp*.*3 | *Brachyteles arachnoides* | 1 | [1] |
| Myrtaceae | *Myrcia splendens* | *Brachyteles arachnoides* | 1 | [11] |
| Myrtaceae | *Myrcia variabilis* | *Brachyteles arachnoides* | 1 | [12] |
| Myrtaceae | *Myrciaria floribunda* | *Brachyteles arachnoides* |  | [18] |
| Myrtaceae | *Myrciaria* sp*.* | *Brachyteles arachnoides, B. hypoxanthus* | | [13], [31] |
| Myrtaceae | *Myrtaceae* | *Brachyteles arachnoides* |  | [26] |
| Myrtaceae | *Myrtaceae* 1 | *Brachyteles arachnoides* |  | [12] |
| Myrtaceae | *Myrtaceae* 2 | *Brachyteles arachnoides* |  | [12] |
| Myrtaceae | *Myrtaceae* 3 | *Brachyteles arachnoides* |  | [12] |
| Myrtaceae | *Myrtaceae* 4 | *Brachyteles arachnoides* |  | [12] |
| Myrtaceae | *Neomithrantes obscura* | *Brachyteles arachnoides* |  | [16] |
| Myrtaceae | *Psidium* aff*. sartorianum* | *Brachyteles arachnoides* | 4 | [11] |
| Myrtaceae | *Psidium cattleianum* | *Tapirus terrestris* | 3 | [21] |
| Myrtaceae | *Psidium guajava* | *Tapirus terrestris* | 4 | [3], [14], [22] |
| Myrtaceae | *Psidium guineense* | *Tapirus terrestris* | 3.41 | [3] |
| Myrtaceae | *Psidium longipetiolatum* | *Brachyteles arachnoides* | 4 | [18], [27] |
| Myrtaceae | *Psidium myrtoides* | *Tapirus terrestris* | 6 | [15] |
| Myrtaceae | *Psidium* sp*.* | *Tapirus terrestris* | 3 | [6], [9] |
| Myrtaceae | *Siphoneugena densiflora* | *Brachyteles arachnoides* |  | [11] |
| Myrtaceae | *Syzygium cumini* | *Tapirus terrestris* | 10 | [3] |
| Olacaceae | *Heisteria silvianii* | *Brachyteles arachnoides* | 12 | [20] |
| Olacaceae | *Tetrastylidium grandifolium* | *Brachyteles hypoxanthus* | 22 | [2] |
| Passifloraceae | *Passiflora* sp*.* 1 | *Tapirus terrestris* | 3.7 | [6] |
| Passifloraceae | *Passiflora* sp*.* 2 | *Brachyteles arachnoides* | 3.7 | [1] |
| Passifloraceae | *Passiflora* sp*.* 3 | *Brachyteles arachnoides* | 3.7 | [1] |
| Phyllanthaceae | *Hieronyma alchorneioides* | *Brachyteles arachnoides* | 2 | [12], [18], [20], [26], [27] |
| Phyllanthaceae | *Hieronyma alchorneoides* | *Tapirus terrestris* | 2 | [26] |
| Phyllanthaceae | *Hieronyma oblonga* | *Brachyteles hypoxanthus* | 2 | [13] |
| Phytolaccaceae | *Phytolacca dioica* | *Brachyteles arachnoides* | 2 | [18], [20], [27] |
| Piperaceae | *Piper* sp*.* | *Tapirus terrestris* | 0.5 | [7] |
| Polygalaceae | *Diclidanthera* sp*.* | *Brachyteles arachnoides* |  | [25] |
| Polygonaceae | *Coccoloba mosenii* | *Brachyteles hypoxanthus* |  | [2] |
| Primulaceae | *Cybianthus coriaceus* | *Brachyteles hypoxanthus* |  | [2] |
| Primulaceae | *Myrcine* sp*.* | *Tapirus terrestris* | 4 | [14] |
| Quiinaceae | *Quiina glaziovii* | *Brachyteles arachnoides* | 9 | [18] |
| Rhamnaceae | *Colubrina glandulosa* | *Tapirus terrestris* | 2 | [14] |
| Rosaceae | *Prunus brasiliensis* | *Brachyteles arachnoides* |  | [19] |
| Rosaceae | *Prunus sellowii* | *Brachyteles arachnoides, B. hypoxanthus* | 8 | [11], [13] |
| Rubiaceae | *Coussarea contracta* | *Brachyteles arachnoides* | 10 | [20] |
| Rubiaceae | *Coussarea triflora* | *Brachyteles hypoxanthus* | 12 | [2] |
| Rubiaceae | *Duroia cf. velutina* | *Brachyteles hypoxanthus* | 4 | [2] |
| Rubiaceae | *Genipa americana* | *Tapirus terrestris* | 8 | [4], [6], [9], [14] |
| Rubiaceae | *Palicourea* sp*.* | *Tapirus terrestris* | 3 | [9] |
| Rubiaceae | *Posoqueria acutifolia* | *Brachyteles arachnoides* | 13 | [26] |
| Rubiaceae | *Posoqueria latifolia* | *Brachyteles arachnoides* | 12 | [20], [18] |
| Rubiaceae | *Posoqueria* sp*.* | *Brachyteles arachnoides* |  | [27] |
| Rubiaceae | *Psychotria mapourioides* | *Brachyteles arachnoides, B. hypoxanthus* | 2 | [26], [2] |
| Rubiaceae | *Psychotria* sp*.* | *Tapirus terrestris* | 3 | [3], [4], [9], [14], [15], [22] |
| Rubiaceae | *Psychotria* sp*.* | *Brachyteles arachnoides* | 3 | [11] |
| Rubiaceae | *Psychotria tenerior* | *Brachyteles arachnoides* | 3 | [11] |
| Rutaceae | *Zanthoxylum* sp*.* | *Tapirus terrestris* | 2 | [14] |
| Salicaceae | *Casearia* sp*.* | *Tapirus terrestris* | 10 | [14] |
| Salicaceae | *Casearia sylvestris* | *Brachyteles arachnoides* | 1.3 | [11] |
| Salicaceae | *Xylosma pseudosalzmanii* | *Brachyteles arachnoides* |  | [19] |
| Sapindaceae | *Allophylus edulis* | *Brachyteles arachnoides* | 4 | [1] |
| Sapindaceae | *Matayba* sp*.* | *Brachyteles hypoxanthus* |  | [2] |
| Sapindaceae | *Paullinia carpopoda* | *Brachyteles arachnoides* | 8 | [1] |
| Sapotaceae | *Chrysophyllum* sp. | *Brachyteles arachnoides* | 7.5 | [26] |
| Sapotaceae | *Chrysophyllum viride* | *Brachyteles arachnoides* | 5 | [12], [18], [20], [26], [27] |
| Sapotaceae | *Diploon cuspidatum* | *Tapirus terrestris* | 14 | [4] |
| Sapotaceae | *Ecclinusa guianensis* | *Tapirus terrestris* | 9 | [4] |
| Sapotaceae | *Micropholis gardneriana* | *Brachyteles arachnoides* | 9 | [26] |
| Sapotaceae | *Pouteria bullata* | *Brachyteles arachnoides* | 9 | [18], [26], [27] |
| Sapotaceae | *Pouteria* sp*.* | *Brachyteles arachnoides, B. hypoxanthus* | 9 | [13], [18], [26] |
| Sapotaceae | *Pouteria* sp*.* | *Tapirus terrestris* | 9 | [4], [8], [9], [26] |
| Sapotaceae | *Pradosia kuhlmannii* | *Brachyteles arachnoides* |  | [27] |
| Sapotaceae | *Sideroxylon obtusifolium* | *Brachyteles arachnoides* | 6 | [11], [12] |
| Schoepfiaceae | *Schoepfia lucida* | *Brachyteles hypoxanthus* |  | [2] |
| Siparunaceae | *Siparuna guianense* | *Brachyteles arachnoides* | 5 | [1], [20] |
| Solanaceae | *Solanum excelsum* | *Brachyteles arachnoides* |  | [11] |
| Solanaceae | *Solanum inaequale* | *Brachyteles arachnoides* | 1 | [11] |
| Solanaceae | *Solanum pseudo-quina* | *Brachyteles arachnoides* | 3 | [26] |
| Solanaceae | *Solanum* sp*.* | *Brachyteles arachnoides* |  | [19] |
| Solanaceae | *Solanum swartzianum* | *Brachyteles arachnoides, B. hypoxanthus* | 1 | [11], [12], [13] |
| Sterculiaceae | *Guazuma ulmifolia* | *Tapirus terrestris* | 1.8 | [9] |
| Styracaceae | *Styrax pohlii* | *Brachyteles arachnoides* | 7 | [16] |
| Symplocaceae | *Symplocos celastrina* | *Brachyteles arachnoides* |  | [11] |
| Symplocaceae | *Symplocos* sp. | *Brachyteles arachnoides, B. hypoxanthus* | | [2], [12], [26] |
| Urticaceae | *Cecropia glaziovi* | *Brachyteles arachnoides* | 1 | [11], [18], [20] |
| Urticaceae | *Cecropia hololeuca* | *Brachyteles hypoxanthus* | 1 | [13] |
| Urticaceae | *Cecropia* sp*.* | *Tapirus terrestris* | 1 | [9] |
| Urticaceae | *Coussapoa microcarpa* | *Tapirus terrestris* | 12 | [26] |
| Urticaceae | *Coussapoa microcarpa* | *Brachyteles arachnoides, B. hypoxanthus* | 12 | [13], [18], [26], [27] |
| Urticaceae | *Coussapoa* sp*.* | *Brachyteles arachnoides* | 12 | [11] |
| Urticaceae | *Pourouma guianensis* | *Brachyteles arachnoides, B. hypoxanthus* | 9 | [13], [20] |
| Urticaceae | *Pourouma guianensis* | *Tapirus terrestris* | 9 | [5] |
| Urticaceae | *Pourouma* sp*.* | *Tapirus terrestris* | 9 | [4], [8] |
| Verbenaceae | *Cytharexylum myrianthum* | *Tapirus terrestris* | 5 | [14] |
| Verbenaceae | *Duranta* sp*.* | *Brachyteles arachnoides* |  | [1] |
| Violaceae | *Rinoreocarpus ulei* | *Tapirus terrestris* | 5 | [9] |
| Vitaceae | *Cissus* sp*.* 1 | *Brachyteles arachnoides* | 7.7 | [1] |
| Vitaceae | *Cissus* sp*.* 2 | *Brachyteles arachnoides* | 7.7 | [1] |

| **Literature cited of Table S2.**   1. Brozek RM (1991) Observações sobre a ecologia alimentar e a dispersão de sementes pelo muriqui (*Brachyteles arachnoides* E. Geoffroy, 1806 - /Cebidae, Primates). Bach Thesis. Universidade Estadual Paulista- Julio de Mesquita Filho: São Paulo. 124 p. 2. Strier KB (1990) New world primates, new frontiers: insights from the wolly spider monkey, or muriqui (*Brachyteles arachnoides*). Int J Primatol 11: 7-19. 3. Bachand M, Trudel OC, Ansseau C, Almeida-Cortez J (2009) Dieta de *Tapirus terrestris* Linnaeus em um fragmento de Mata Atlântica do Nordeste do Brasil. R Bras Bioci 7: 188-194. 4. Hibert F, Sabatier D, Andrivot J, Scotti-Saintagne C, Gonzalez S, Prévost M-F, Grenand P, Chave J, Caron H, Richard-Hansen C (2011) Botany, genetics and ethnobotany:a crossed investigation on the elusive tapir’s diet in French Guiana. PLoS ONE 6: e25850. doi:10.1371/journal.pone.0025850 5. Salas LA, Fuller TK (1996) Diet of the lowland tapir (*Tapirus terrestris* L) in the   Tabaro River Valley, southern Venezuela. Can J Zool 74: 1444–1451.   1. Fragoso JMV, Huffman JM (2000) Seed-dispersal and seedling recruitment patterns by the last Neotropical megafaunal element in Amazonia, the tapir. J Trop Ecol 16: 369–385. 2. Henry O, Feer F, Sabatier D (2000) Diet of the lowland tapir (*Tapirus terrestris*) in   French Guiana. Biotropica 3: 364–368.   1. Montenegro O (2004) Natural licks as keystone resources for wildlife and people   in Amazonia. PhD Dissertation. University of Florida: Florida. 145 p.   1. Tobler MW (2008) The ecology of the lowland tapir in Madre de Dios, Peru:   using new technologies to study large rainforest mammals. PhD Dissertation.  Texas A&M University: College StationTexas. 132 p.   1. Bodmer RE (1991) Influence of digestive morphology on resource partitioning in   Amazonian ungulates. Oecologia 85: 361–365.   1. Carvalho Jr O (1996) Dieta, padrões de atividades e de agrupamento do mono-carvoeiro (*Brachyteles arachnoides*) no Parque Estadual Carlos Botelho – SP. MSc Thesis, Universidade Federal do Pará: Belém. 56p. 2. Moraes PLR (1992) Dispersão de sementes pelo mono-carvoeiro (*Brachyteles arachnoides* E. Geoffroy, 1806) no Parque Estadual de Carlos Botelho. *2° Congresso Nacional sobre Essências Nativas*. Revista do Instituto Florestal, Sao Paulo: 1193-1198. 3. Teixeira RNC (2006) A importância de remanescentes de Mata Atlântica na ocorrência de muriquis (*Brachyteles hypoxanthus* E. Geoffroy) no estado do Espírito Santo. MSc Thesis. Universidade Federal de Viçosa: Viçosa- MG. 87p. 4. Tófoli CF (2006) Frugivoria e dispersão de sementes por *Tapirus terrestris* (Linnaeus, 1758) na paisagem fragmentada do Pontal do paranapanema, São Paulo. MSc Thesis. Universidade de São Paulo: São Paulo. 89 p. 5. Talamoni SA, Assis MAC (2009) Feeding habit of the Brazilian tapir, *Tapirus*   *terrestris* (Perissodactyla: Tapiridae) in a vegetation transition zone in southeastern  Brazil. Zoologia 26: 251–254.   1. Martins MM (2009) Lianas as a food resource for brown howlers (*Alouatta guariba*) and southern muriquis (*Brachyteles arachnoides*) in a forest fragment. Animal Biodiversity and Conservation 32: 51-58. 2. Torres CA (1983) An ecological study of the primates of Southeastern Brazil, with a reappraisal of *Cebus apella* races. PhD dissertation. University of Edinburg: Edinburg. 337 p. 3. Petroni L (2000) Caracterização da área de uso e dieta do mono-carvoeiro (*Brachyteles arachnoides*, Cebidae- Primates) na Mata Atlântica, Serra de Paranapiacaba, SP. PhD dissertation. Universidade de São Paulo: São Paulo. 166p. 4. Pereira LCM (2006) Área de vida e padrões de deslocamento de (*Brachyteles*   *Arachnoides* E. Geoffroy, 1806 Primates: Atelinae) em um fragmento florestal no Município de Castro, Estado do Paraná, Brasil. Universidade Federal do Paraná: Curitiba. 120 p.   1. Izar P (2008) Dispersão de sementes por *Cebus nigritus* e *Brachyteles arachnoides* em área de Mata Atlântica, Parque Estadual Intervales, SP. A Primatologia no Brasil - 9 (S.F. Ferrari & J. Rímoli, Eds.) Aracaju, Sociedade Brasileira de Primatologia, Biologia Geral e Experimental – UFS. Pp. 8-24. 2. Santos LGRO, Machado LCP, Tortato MA, Falkenberg DB, Ho¨ tzel MJ (2005)   Diet of tapirs (*Tapirus terrestris*) introduced in a salt marsh area of the Baixada do  Massiambu, State Park of the Serra do Tabuleiro - Santa Catarina, South of  Brazil. The Newsletter of the IUCN/SSC Tapir Specialist Group (TSG) 14:  22–27.   1. Galetti M, Keuroghlian A, Hanada L, Morato NI (2001) Frugivory and seed   dispersal by the lowland tapir (*Tapirus terrestris*) in Southeast Brazil . Biotropica  33: 723–726.   1. Giombini MI, Bravo SP, Martinez MF (2009) Seed dispersal of the palm *Syagrus*   *romanzoffiana* by tapirs in the semi-deciduous atlantic forest of Argentina.  Biotropica 41: 408–413.   1. Olmos F, Pardini R, Boulhosa RLP, Burgi R, Morsello C (1999) Do tapirs steal   food from palm seed predators or give them a lift? Biotropica 31: 375–379.   1. Martins MM (2006) Comparative seed dispersal effectiveness of sympatric *Alouatta guariba* and *Brachyteles arachnoides* in Southeastern Brazil. *Biotropica* 38: 57-63. 2. Bueno R (2010) Frugivoria e efetividade de dispersão de sementes dos últimos grandes frugívoros da Mata Atlântica: a anta (*Tapirus terrestris*) e o muriqui (*Brachyteles arachnoides*). MSc Thesis. Universidade Estadual Paulista: Rio Claro. 67 p. 3. Petroni L (1993) Aspectos da ecologia e comportamento do mono-carvoeiro (*Brachyteles arachnoides-* E. Geoffroy, 1806- Cebidae, Primates) na Fazenda Intervales, Serra de Paranapiacaba, São Paulo. MSc Thesis. Pontifícia Universidade Católica do Rio Grande do Sul: Porto Alegre. 78p. 4. Zórzi BT (2009) Frugivoria por *Tapirus terrestris* em três regiões do Pantanal, Brasil. MSc Thesis. Universidade Federal do Mato Grosso do Sul: Campo Grande. 43 p. 5. Mourthé IMC, Strier KB, Boubli JP (2008) Seed Predation of *Mabea fistulifera* (Euphorbiaceae) by Northern Muriquis (*Brachyteles hypoxanthus*). Neotropical Primates 15:40-45. 6. Martins MM (2008) Fruit diet of *Alouatta guaraba* and *Brachyteles arachnoides* in southeastern Brazil: comparison of fruit type, color, and seed size. Primates 49:1-8. 7. Milton KS (1984) Habitat, diet and activity patterns of free-ranging woolly spider monkeys (*Brachyteles arachnoides* E. Geoffroy 1806). Int J Primatol 5: 491-514. 8. Talebi M, Bastos A, Lee PC (2005) Diet of Southern muriquis in continuous Brazilian Atlantic Forest. Int J Primatol 26: 1175-1187. 9. Angiosperm Phylogeny Group (2009) An updated of the Angiosperm Phylogeny Group classification for orders and families of flowering plants. Botanical Journal of Linnean Society 161: 105-121. |  |  |  |  |  |
| --- | --- | --- | --- | --- | --- |
